# A revised concept of moyamoya vasculopathy: developmental origins and genetic insights

**DOI:** 10.3389/fneur.2025.1653558

**Published:** 2025-09-10

**Authors:** Takahiro Ota

**Affiliations:** Department of Neurosurgery, Tokyo Metropolitan Tama Medical Center, Tokyo, Japan

**Keywords:** moyamoya vasculopathy, neural crest, neurocristopathy, RNF213, *RNF213* vasculopathy

## 1 Introduction

Moyamoya vasculopathy (MMV) is a cerebrovascular condition characterized by progressive stenosis of the intracranial internal carotid arteries (ICAs) and their proximal branches, often resulting in stroke. The hallmark of MMV is progressive intracranial ICA stenosis and the formation of collateral vessels known as moyamoya vessels. Patients presenting with characteristic MMV and known concomitant associated conditions are classified as having moyamoya syndrome (MMS), whereas those with no identifiable risk factors are diagnosed with moyamoya disease (MMD) (1).

The 2022 revised diagnostic criteria by the Japanese Research Committee on Moyamoya Disease introduced significant updates, including the recognition of both unilateral and bilateral cases, and the inclusion of concomitant hyperthyroidism as MMD (2). However, confusion with respect to naming remains owing to inconsistent terminology in the previous literature and the inherent difficulty in establishing definitive diagnostic boundaries.

This study proposes a revised concept of MMV within the framework of steno-occlusive disease of the circle of Willis, incorporating developmental and genetic insights. This revised concept may clearly explain the relationship between MMV and steno-occlusive disease of the circle of Willis, and facilitate the understanding of the actual presentations of MMV, which cannot be explained by the present concept of MMD/MMS.

## 2 MMD and MMS

MMD and MMS may have different disease prognosis, have different imaging features using high resolution MRI vessel wall imaging, and needs different treatment strategies (3, 4). The distinction between MMD and MMS is clinically important but is often ambiguous due to overlapping features. The differences between MMD and MMS concerning their pathogenesis have not been clearly elucidated. There are several points in the overlap between the two categories, which are discussed as follows. Radiologically, both MMD and MMS demonstrate progressive stenosis of the ICA and proximal portions of the anterior cerebral artery (ACA) and middle cerebral artery (MCA), along with the development of basal moyamoya vessels. Because of this similarity, imaging alone is insufficient for a definitive diagnosis. Clinically, both entities can present with ischemic events, hemorrhages, or transient ischemic attacks and can affect both pediatric and adult populations (1, 5). Recent discoveries in basic research have revealed shared genetic and pathophysiological mechanisms between MMD and MMS. Mutations in the *RNF213* gene have been identified in MMD and a subset of MMS cases (6). This finding suggests a common genetic predisposition. Therefore, it is reasonable to consider MMD and MMS as single disease concepts—MMV—without the need for strict differentiation.

## 3 Relationship between MMV and *RNF213* vasculopathy

MMV is increasingly recognized as a genetically influenced cerebrovascular arteriopathy, with strong evidence implicating mutations in the *RNF213* gene—particularly the East Asian-specific variant p.R4810K (c.14429G>A). This variant is present in over 80% of East Asian patients with familial MMD and approximately 50% of sporadic cases, suggesting a central role in disease susceptibility in this population (6, 7). However, it is also found in approximately 5% of patients with non-cardioembolic stroke and 2% of healthy controls (8).

Although the full range of the biological functions of *RNF213* remains under investigation, it is known to influence vascular remodeling, angiogenesis, and endothelial cell homeostasis (9). Interestingly, not all individuals carrying *RNF213 p.R4810K* mutations develop MMV—especially heterozygous carriers—who often exhibit low disease penetrance. This supports a multifactorial disease model in which genetic susceptibility alone is insufficient to cause MMV, and additional environmental or physiological triggers are required (10).

The term “*RNF213*-associated vasculopathy” has been proposed to encompass the broader vascular spectrum linked to these mutations. In addition to classic intracranial arterial stenosis, affected individuals may exhibit extracranial vascular abnormalities such as coronary artery disease, renal artery stenosis, pulmonary artery stenosis, and peripheral arteriopathy. These findings suggest that systemic vascular fragility extends beyond the central nervous system (11, 12).

This broader understanding suggests that MMV represents a central nervous system-specific phenotype within the spectrum of *RNF213*-associated vasculopathy.

## 4 Multifunctional roles of *RNF213*: vascular and anti-infectious functions

RNF213 is a multifunctional protein with functions that extend beyond vascular development, immune regulation, and host defense (13). In the vascular context, *RNF213* modulates endothelial cell migration, angiogenic sprouting, and arterial remodeling. A deficiency or mutation leads to impaired vascular patterning and increased fragility, contributing to the steno-occlusive pathology of MMV. It also influences the vascular smooth muscle cell phenotype and lipid metabolism, implicating it in systemic vasculopathy.

*RNF213* plays a role in innate immunity and antimicrobial defense. Its role in tagging bacterial components via ubiquitination and modulating membrane dynamics suggests that it functions as a part of the cell-autonomous immune machinery (14, 15). MMV has also been reported in patients with bacterial and viral infections. Immune-related functions may provide insight into the triggers of MMV. In genetically susceptible individuals, immune activation owing to infection, inflammation, or autoimmune conditions may catalyze disease onset. Thus, *RNF213* can be considered a molecular hub at the intersection of vascular biology, immunity, and inflammation, reinforcing its central role in the multifactorial pathogenesis of MMV (10).

## 5 Vascular cephalic neurocristopathy and MMV

The concept of vascular cephalic neurocristopathy offers a developmental and embryological framework for understanding MMV (16, 17). Cephalic neural crest cells migrate into the pharyngeal arches and frontonasal processes, becoming the forehead, the middle of the nose, and the primary palate. The cephalic neural crest provides mesenchymal cells to the arteries in the cardio- and cerebrovascular regions, whereas the endothelium of all the blood vessels in the body, including the brain, originate from the mesoderm (18). Disruptions in their migration, differentiation, or maturation can impair the structural integrity of the cerebral arteries, especially those within the territory of the circle of Willis.

Vascular cephalic neurocristopathy posits that MMV results from the segmental vulnerability of neural crest-derived vessels, particularly those in the embryological anterior circulation around the circle of Willis, such as the ICA, ACA, MCA, and posterior cerebral artery (16, 17, 19). These arteries may be developmentally predisposed to pathological remodeling, especially under additional genetic or environmental stressors. However, the contribution of neural crest cells to MMV development has not been directly proven.

This model also helps to explain the regional specificity of MMV and supports the notion that MMV is a congenital or early-onset vascular disorder with a neurodevelopmental basis. The combination of embryological susceptibility and acquired molecular triggers (e.g., *RNF213* dysfunction and immune activation) aligns with the multifactorial nature of MMV and offers a unifying explanation for its selective distribution and clinical variability.

This developmental susceptibility may partly explain the selective involvement of the arteries of the circle of Willis in MMV.

## 6 Twig-like MCAs

Twig-like MCAs have been reported to present with angiographic imaging findings similar to those of MMV. It is considered a rare vascular anomaly characterized by a network of fine arterial channels replacing the main trunk of the MCA. However, the concept of a twig-like MCA has not yet been established, and its pathogenesis is controversial (20). Twig-like MCA is recognized as a secondary collateral formation following proximal MCA hypoplasia or occlusion, placing it within the broader category of the steno-occlusive diseases affecting the circle of Willis. The presence of *RNF213* p.R4810K mutations in some twig-like MCA cases (21) suggests that this entity may belong within the broader steno-occlusive disease of the circle of Willis spectrum, particularly among genetically predisposed individuals.

## 7 Discussion

The conception of MMV has already been used for years and diagnosis of MMV requires both progressive steno-occlusion of the arteries around the circle of Willis and the presence of characteristic basal collateral vessels. However, it is difficult to precisely define moyamoya vessels on angiography, making it challenging to distinguish MMV from other steno-occlusive diseases based solely on imaging. Autoimmune diseases, infections/inflammation, radiation exposure, and vasculitis are among the underlying diseases that cause MMV; however, even if the etiology is unclear at present, it may become clear in the future as research progresses. In a real-world clinical cohort, surgical revascularization was effective in both MMD and MMS, suggesting that an accurate classification, while relevant to understanding pathogenesis, may not always be necessary for guiding treatment decisions (22). In practice, treatment decisions often depend more on the severity and progression of MMV than on a strict etiological classification. This has led to increased support for viewing MMV as a single entity that accommodates genetic, environmental, and developmental contributions to disease expression.

The *RNF213* p.R4810K variant is attracting attention for its involvement in the pathogenesis of MMV, but not all individuals with the *RNF213* p.R4810K variant develop MMV, indicating low penetrance and suggesting that additional factors are required to trigger disease onset. The “two-hit hypothesis” posits that a genetic predisposition (*RNF213* mutation) interacts with secondary insults such as inflammation, radiation exposure, or autoimmune processes to manifest MMV (3, 4). However, recent findings have also implicated *RNF213* in immune regulation and antimicrobial defense, complicating its role as a purely genetic first hit. Instead, the concept of vascular cephalic neurocristopathy may offer a more appropriate framework for understanding disease initiation. This model posits that embryological disruption in the neural crest-derived vascular structures of the embryological anterior circulation creates vulnerable vascular substrates (16, 17). Within this context, RNF213 dysfunction may serve as a second or one of the multiple hits, along with autoimmune disorders, infections/inflammation, endothelial progenitor cells, and hemodynamic stress, which trigger MMV onset in a predisposed vascular milieu.

MMV is characterized by arterial steno-occlusive changes around the circle of Willis. While MMV and steno-occlusive disease of the circle of Willis are related, they are not synonymous. Steno-occlusive diseases around the circle of Willis can result from various etiologies, including atherosclerosis, vasculitis, infection, sickle cell disease, Down syndrome, radiation-induced arteriopathy, neurofibromatosis type I, and genetic syndromes. Unlike MMV, these conditions do not necessarily involve the formation of basal collaterals or moyamoya vessels. Furthermore, they may be unilateral, asymmetrical, or components of a broader systemic disorder.

Traditionally, diseases such as MMV and atherosclerotic intracranial stenosis have been treated as distinct clinical entities because their pathologies are essentially different. In contrast, in the early stages of MMV, some patients present with only steno-occlusive changes around the circle of Willis without the development of moyamoya vessels. As the disease progresses, moyamoya vessels may appear, making the initial diagnosis of MMV uncertain.

**Figure 1:**
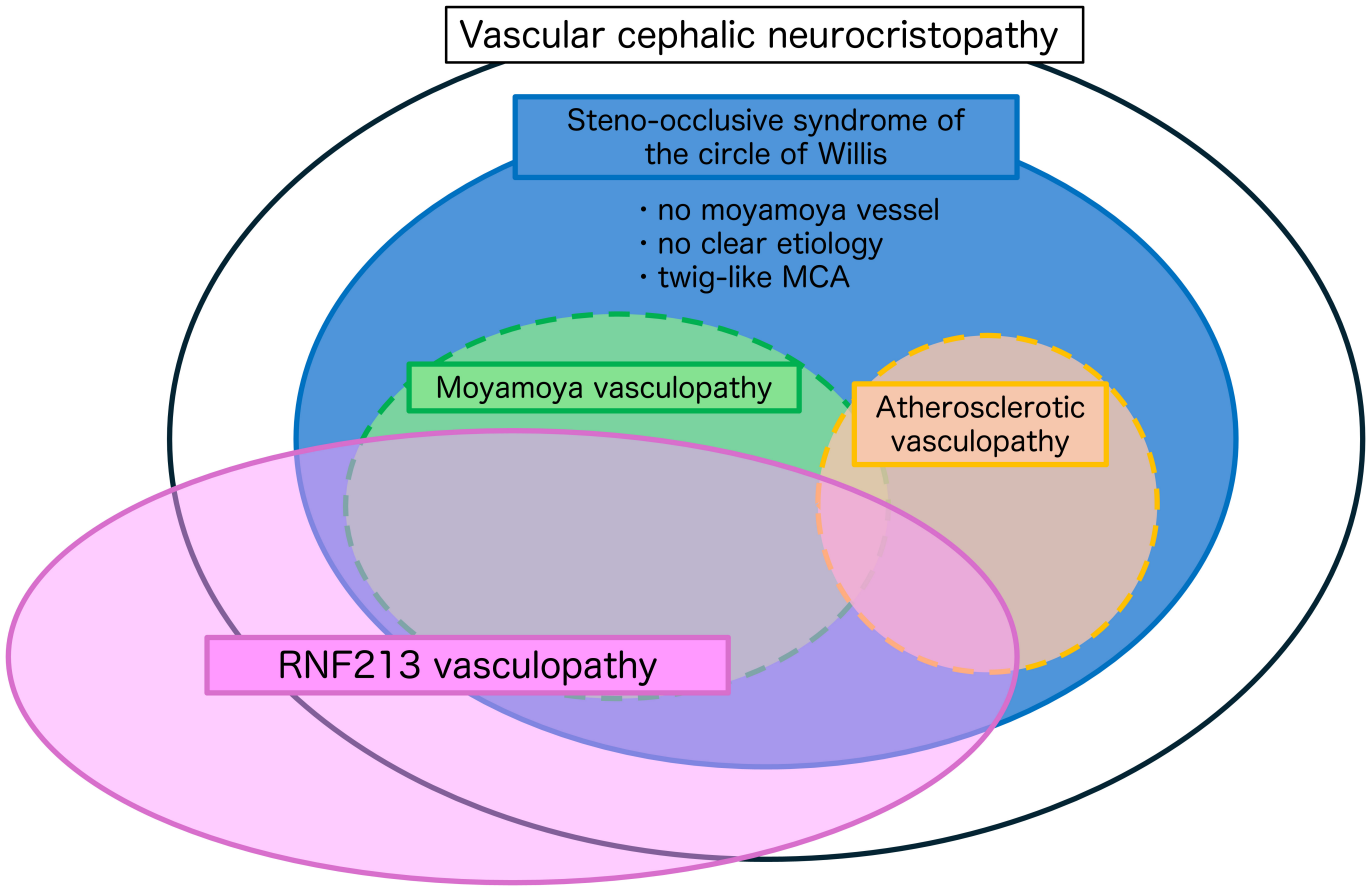
Conceptual framework of steno-occlusive disease of the circle of Willis. This schematic illustrates the overlapping pathophysiological spectra of intracranial steno-occlusive diseases centered on the circle of Willis. The large black oval represents the proposed concept of vascular cephalic neurocristopathy, encompassing developmental and genetic influences affecting the cerebral arteries. The blue ellipse defines steno-occlusive syndrome of the circle of Willis, which includes cases with no definitive moyamoya vessels, no clear etiology, or atypical presentations, such as twig-like MCA. Moyamoya vasculopathy (green) and atherosclerotic vasculopathy (orange) are classically distinct, but partially overlapping diseases within this spectrum. The pink ellipse denotes RNF213 vasculopathy, illustrating a shared genetic predisposition across multiple phenotypes, including moyamoya vasculopathy, a subset of atherosclerotic stenosis, and other unclassified steno-occlusive changes. This framework supports the idea that steno-occlusive disease of the circle of Willis represents a broad-spectrum entity, which may aid in the classification of diagnostically ambiguous cases and facilitate a better understanding of disease mechanisms and treatment strategies.

However, several observations discussed above support the notion that they may represent a spectrum of broader pathophysiological processes centered around the circle of Willis, such as anatomical predilection (*vascular cephalic neurocristopathy)*, genetic overlap (*RNF213 vasculopathy*), and clinical ambiguity. Taken together, these considerations support the utility of a unifying framework, steno-occlusive disease of the circle of Willis, to encompass this spectrum of cerebrovascular pathologies. By conceptualizing steno-occlusive disease of the circle of Willis as a broad-spectrum entity, we can better classify and contextualize previously unclassifiable or diagnostically challenging cases of arterial steno-occlusive disease in this region, such as cases without typical moyamoya vessels, without a clear etiology, or the so-called twig-like MCA (20) (Figure 1). This approach facilitates a more comprehensive understanding of the underlying common pathogenesis and contributes to informed therapeutic decision-making. Nevertheless, it is important to note the limitations, specifically the lack of direct experimental evidence (e.g., from animal models) to firmly establish the vascular cephalic neurocristopathy hypothesis and how specific *RNF213* variants affect the development of MMV. Therefore, a specific evaluation of the usefulness of this concept in clinical cases is required.

In conclusion, conceptualizing MMV as a subgroup of steno-occlusive diseases of the circle of Willis may aid in the classification of diagnostically ambiguous cases and facilitate a better understanding of the disease mechanisms and treatment strategies. Future diagnostic and therapeutic approaches should move beyond rigid classifications and toward a more integrative, pathophysiology-based understanding of steno-occlusive diseases in the circle of Willis. Personalized care benefits from multidisciplinary collaboration, genetic insights, and the recognition of this disease as a dynamic, multifactorial disease spectrum.
